# Updates from the SPADnet project (fully digital, scalable and networked photonic component for Time-of-Flight PET applications)

**DOI:** 10.1186/2197-7364-1-S1-A11

**Published:** 2014-07-29

**Authors:** Edoardo Charbon, Claudio Bruschini, Chockalingam Veerappan, Leo HC Braga, Nicola Massari, Matteo Perenzoni, Leonardo Gasparini, David Stoppa, Richard Walker, Ahmed Erdogan, Robert K Henderson, Steve East, Lindsay Grant, Balázs Jatekos, Ferenc Ujhelyi, Gábor Erdei, Emöke Lörincz, Luc André, Laurent Maingault, Vincent Reboud, Loick Verger, Eric Gros d’Aillon, Péter Major, Zoltán Papp, Gábor Németh

**Affiliations:** Delft University of Technology, Delft, The Netherlands; Ecole Polytechnique Fédérale de Lausanne (EPFL), Lausanne, Switzerland; Fondazione Bruno Kessler (FBK), Trento, Italy; University of Edinburgh, Edinburgh, UK; STMicroelectronics (R&D) Ltd, Edinburgh, UK; Budapest University of Technology and Economics (BME), Budapest, Hungary; CEA - LETI, Grenoble, France; Mediso Medical Imaging Systems Ltd, Budapest, Hungary

SPADnet is aimed at a new generation of fully digital, scalable and networked photonic components to enable large area image sensors, with primary target gamma-ray and coincidence detection in (Time-of-Flight) PET. The SPADnet photonic module, which lies at the heart of the concept, is built around an array of tessellated single-photon TSV sensor chips, manufactured in standard CMOS technology. The resulting sensor tile is connected on the back to an FPGA-based data processing and communication unit, whereas its front size is glued to scintillator crystals. The resulting modules are then connected in a token ring structure to form the actual PET system. Coincidence detection occurs directly in the ring itself, in a differed and distributed manner to ensure scalability.

We have fabricated and tested the first version of the SPADnet photosensor, a fully digital CMOS SiPM with 8×16 pixels individually capable of photon time stamping and energy accumulation, together with the corresponding sensor tiles. The sensor also provides a real-time output of the total detected energy at up to 100Msamples/s and on-chip discrimination of gamma events. These events can then be routed to the SPADnet ring network, which operates at 2 Gbps providing real-time processing and coincidence determination; this architecture simplifies the construction of the overall system and allows the scaling of the system to larger arrays of detectors. This may result in better and faster image reconstruction.

SPADnet will not only impact PET scalability but also performance robustness and cost; another advantage is the capability of being compatible with magnetic resonance imaging (MRI), thus prompting advances in multimodal imaging and medical diagnostics as a whole.

SPADnet is being designed with scalability in mind, with the idea of being able to redeploy at reduced effort the SPADnet photonic module in other configurations such as brain PET.
